# Exposure to secondhand smoke and asthma severity among children in Connecticut

**DOI:** 10.1371/journal.pone.0174541

**Published:** 2017-03-31

**Authors:** Jessica P. Hollenbach, Elizabeth D. Schifano, Christopher Hammel, Michelle M. Cloutier

**Affiliations:** 1 Asthma Center, Connecticut Children’s Medical Center, Hartford, Connecticut, United States of America; 2 Department of Pediatrics, University of Connecticut School of Medicine, Farmington, Connecticut, United States of America; 3 Department of Statistics, University of Connecticut, Storrs, Connecticut, United States of America; 4 University of Connecticut School of Medicine, Farmington, Connecticut, United States of America; Cincinnati Children's Hospital Medical Center, UNITED STATES

## Abstract

**Objective:**

To determine whether secondhand smoke (SHS) exposure is associated with greater asthma severity in children with physician-diagnosed asthma living in CT, and to examine whether area of residence, race/ethnicity or poverty moderate the association.

**Methods:**

A large childhood asthma database in CT (Easy Breathing) was linked by participant zip code to census data to classify participants by area of residence. Multinomial logistic regression models, adjusted for enrollment date, sex, age, race/ethnicity, area of residence, insurance type, family history of asthma, eczema, and exposure to dogs, cats, gas stove, rodents and cockroaches were used to examine the association between self-reported exposure to SHS and clinician-determined asthma severity (mild, moderate, and severe persistent vs. intermittent asthma).

**Results:**

Of the 30,163 children with asthma enrolled in Easy Breathing, between 6 months and 18 years old, living in 161 different towns in CT, exposure to SHS was associated with greater asthma severity (adjusted relative risk ratio (aRRR): 1.07 [1.00, 1.15] and aRRR: 1.11 [1.02, 1.22] for mild and moderate persistent asthma, respectively). The odds of Black and Puerto Rican/Hispanic children with asthma being exposed to SHS were twice that of Caucasian children. Though the odds of SHS exposure for publicly insured children with asthma were three times greater than the odds for privately insured children (OR: 3.02 [2.84,3,21]), SHS exposure was associated with persistent asthma only among privately insured children (adjusted odds ratio (aOR): 1.23 [1.11,1.37]).

**Conclusion:**

This is the first large-scale pragmatic study to demonstrate that children exposed to SHS in Connecticut have greater asthma severity, clinically determined using a systematic approach, and varies by insurance status.

## Introduction

Forty percent of children in the United States are exposed to secondhand smoke (SHS) [1]. Despite the Surgeon General’s and National Asthma Education and Prevention Program (NAEPP) recommendations to identify and avoid SHS, 53% of children with asthma remain exposed[2]. In 2004 Connecticut enacted the Clean Indoor Air Act. Despite this law, in 2013 38% of Connecticut youth were still exposed to SHS in an indoor or outdoor public place[3]. Children with asthma exposed to SHS are at increased risk for more severe symptoms and for asthma exacerbations as compared to children not exposed to SHS[4–7]. Studies also suggest that children with asthma exposed to SHS may experience greater asthma severity, but these studies had small sample sizes and did not systematically determine asthma severity[8] or preferentially enrolled children with greater severity[9, 10]. Asthma prevalence is associated with socioeconomic disparity with people of low income and people of color disproportionately affected and experiencing higher rates of persistent disease. Environmental exposures, such as SHS, are higher in urban communities and may contribute to this disparity[11].

Asthma severity is the intrinsic intensity of the disease and according to the 2007 NAEPP guidelines is determined using a combination of clinical history and spirometry [12]. The Easy Breathing Survey translates the NAEPP guidelines into a format that can be implemented in primary care offices[12], and guides clinicians to better manage childhood asthma by including an assessment of asthma risk factors, such as SHS and other environmental exposures. Clinicians then systematically determine asthma severity using 4 validated clinical questions and create a severity-specific treatment plan for their patient[13]. Ninety-five percent of children with asthma enrolled in Easy Breathing have received a written asthma treatment plan, and 94% of these plans adhere to guidelines for prescription of controller therapy for persistent disease[14].

The goal of this study was to determine the effect of exposure to SHS on systematically determined asthma severity in a large cohort of children enrolled in a disease management program in CT[15]. The robust Easy Breathing data provide the opportunity to examine the association between real-world exposure to caregiver-reported SHS and guideline-determined asthma severity in children, which has previously elicited discordant results[10, 16–18], while controlling for well-known risk factors. To explore this relationship we used the CT Easy Breathing database composed of over 120,000 children, of whom 32,691 have physician-confirmed asthma. The Easy Breathing database contains information about asthma severity, risk factors such as eczema and exposure to cockroach, rodents, gas stove and SHS, and sociodemographic factors such as race/ethnicity, area of residence, and type of health insurance. We also sought to examine whether subpopulations of children with asthma are similarly affected by SHS exposure given observed racial, ethnic, geographic and socioeconomic disparities in asthma rates and morbidity[19, 20].

## Materials and methods

### Sources of data

The Easy Breathing Program has been described previously[21, 22]. This study is a secondary analysis of all children with a documented asthma severity (n = 30,163) enrolled in the Easy Breathing program in CT between April 1, 1998 and September 30, 2013. Clinicians were encouraged to screen all patients in their practice for asthma with the Easy Breathing Survey. The criteria used to suggest an asthma diagnosis have been previously described[13] and included recurrent episodes of wheezing, nocturnal cough, exercise impairment, and the persistence of a cough associated with an upper respiratory tract infection. Using the Easy Breathing Survey as a guide, clinicians diagnosed asthma based on additional available data and clinical judgment. Demographic information including age, sex, race/ethnicity, town of residence, insurance type, history of eczema, family history of asthma, exposure to SHS more than 2 times per week, and exposure to other known asthma triggers were self-reported from the Easy Breathing Survey. Asthma severity was obtained from the Provider Assessment which asks four validated questions (Table 1)[15] and classified as intermittent, mild, moderate or severe persistent according to the NAEPP-EPR3 recommendations. This study was approved by the Connecticut Children’s Institutional Review Board under the Easy Breathing program. Parents/guardians provided written informed HIPAA consent on behalf of the child.

**Table 1:** Provider Assessment from Easy Breathing Survey used by Clinicians to Determine Asthma Severity.

Clinical symptom	Frequency			
Frequency of episodes of cough, wheeze, shortness of breath (daytime)	≤2x/wk	>2x/wk <daily	Daily	Continuously
Frequency of nighttime symptoms	≤2x/mo	>2x/mo	>1x/wk	>4x/wk
Exercise impairment (even with pre-treatment with beta-agonist)	None	Occasionally	Some	Always
School absenteeism for asthma past year (days/month)	< 3	3–5	6–8	>8
**Asthma severity is:**	**Intermittent**	**Mild Persistent**	**Moderate Persistent**	**Severe Persistent**

SHS exposure was dichotomized into Yes or No based on a response to the following question on the Easy Breathing Survey: *Is your child exposed to the following more than 2 times/week? Cigarette or Cigar smoke*.

**Fig 1:**
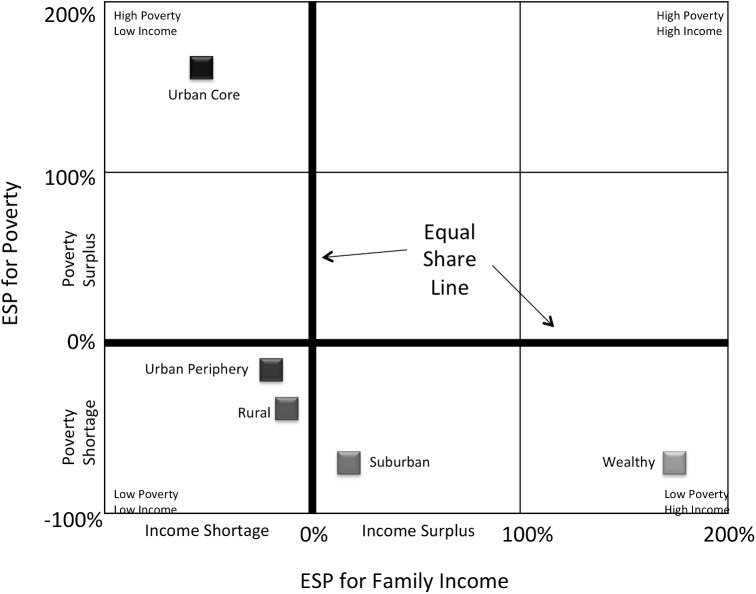
The 5 Connecticut socioeconomic status (SES) categories demonstrating the equal share percentage (ESP) for family income and poverty as of 2000. Town of residence (by participant’s zip code) was classified according to the 5 Connecticuts study as urban core, urban periphery, suburban, wealthy, or rural as proxies for area of residence. These proxies were determined by combining town-level population density, median family income, and percent of residents living in poverty (defined as the percentage of population below the 100% poverty threshold) The equal share line (where ESP = 0%) marks where the share of a variable does not differ from the statewide average. *Adapted with permission from The Five Connecticuts report Figure 7*.

To evaluate the effect of area of residence on asthma severity, town of residence (by participant’s zip code) was classified according to the Five Connecticuts study as urban core, urban periphery, suburban, wealthy, or rural as proxies for area of residence[23]. These proxies for socioeconomic advantage/disadvantage were determined by combining town-level population density, median family income, and percent of residents living in poverty (defined as the percentage of population below the 100% poverty threshold) (Fig 1). Importantly, race/ethnicity was not used to determine group membership. Because there were relatively few people with asthma sampled from wealthy areas, this category was combined with the suburban category and classified as “suburban/wealthy”.

### Covariates

We included covariates previously associated with increased risk of persistent asthma and/or with risk for SHS exposure. Age at enrollment and year of enrollment were treated as continuous variables. Race/ethnicity was self-reported as: African American, Asian or Pacific Islander, Caribbean/Virgin Islander, White/Caucasian, Hispanic/Mexican, Hispanic/Puerto Rican, Hispanic/Cuban, Hispanic/Other, and Other. Because the race/ethnicity of the Other respondents was not known, this category was not included in any analyses. Participants endorsing African American and Caribbean/Virgin Islander were combined into Black and all non-Puerto Rican Hispanics were combined into Hispanic. The Hispanic category was subdivided into Puerto Rican and Hispanic/non-Puerto Rican based on the contribution of African ancestry to, on average, higher asthma morbidity and reduced lung function in Puerto Rican children[24–26]. Other potential confounders included sex, family history of asthma, type of health insurance, history of eczema, and exposure to cats, dogs, cockroaches, gas stove, and rodents. Individual household income, as a measure of socioeconomic status (SES), was not included as it is not collected on the Easy Breathing Survey. However, town-level median household income is accounted for in the Five Connecticut’s proxy designations.

### Statistical analysis

The association of SHS and asthma severity (mild, moderate, and severe persistent vs. intermittent) was assessed by multinomial logistic regression analysis and presented as relative risk ratio (RRR) or as RRR adjusted (aRRR) for potential confounders and 95% confidence intervals (95% CI). The ordinal logistic regression model was also considered, but ultimately not used as the assumption of proportional odds was not satisfied. We report aRRR in the sense that the ratio of probabilities, i.e., the probability of one persistent outcome category (mild, moderate, or severe) over the probability of the intermittent category, is often referred to as relative risk; exponentiated regression coefficients are thus interpreted as relative risk ratios for a unit change in the predictor variable. To assess ordinal association of two variables, we used the Gamma (γ) measure of association. Analyses were adjusted for enrollment date, sex, age, race/ethnicity, family history of asthma, area of residence, type of insurance (Medicaid or private), eczema status, and exposure to dogs, cats, rodents, cockroaches and gas stoves.

We evaluated collinearity of the risk factors using a weighted regression with the intercept-adjusted collinearity diagnostics provided in SAS proc reg, where the weights were calculated from a binary logistic regression for intermittent vs persistent asthma. We additionally computed pairwise polyserial, polychoric or Pearson correlations of predictors as appropriate. For the purposes of correlation analysis, Race/Ethnicity was dichotomized as Caucasian vs Non-Caucasian and Area of Residence was ordered according to the Five Connecticuts (Fig 1) as Urban Core, Urban Periphery, Rural, Suburban/Wealthy.

Interactions between exposure to SHS and confounders such as sex, age, race/ethnicity, insurance type, and area of residence were tested given that the relationship between asthma severity and exposure to SHS may vary between categories of these characteristics. P-values for the tested interactions with SHS are provided (see S6 Table). Because of a significant interaction between SHS exposure status with insurance status, but not between SHS and the other confounders, we conducted stratified analyses by insurance status. Due to the smaller sample sizes, the association of SHS and asthma severity in stratified analyses was assessed by binary logistic regression analysis (persistent vs intermittent) and are presented as odds ratios (OR) or as OR adjusted (aOR) for potential confounders with 95% CI. Covariate associations with SHS were also examined with binary logistic regression and reported similarly. All results are reported using multiple imputation (MI)[27] to handle missing values in the predictor variables unless otherwise noted. Additional sensitivity analyses and complete-case analysis were also conducted; see Supplemental Material (S1 Methods and Tables in S1–S5 Tables). Note that multicollinearity was assessed prior to multiple imputation (i.e., with complete-case analysis), noting that the deletion of the missing data in this step reduces the sample size and produces a more homogenous sample so that collinearity may seemingly be worse in the reduced dataset than in the entire sample[28]. All analyses were performed with SAS version 9.4 (SAS Institute, Cary NC). A p value <0.05 was considered statistically significant unless otherwise noted.

## Results

Between April 1, 1998 and September 30, 2013, 120,854 children between six months and 18 years of age were surveyed in the Easy Breathing program in CT and 32,691 (27%) had physician-confirmed asthma. Asthma severity was documented in 30,163 children (92% with asthma) who comprise the analytic cohort for this study (Table 2). The demographics of the children with asthma in Easy Breathing are similar to the demographics of children in Connecticut[29], with comparable overall rates of exposure to SHS (23% vs 22% respectively)[30]. Children with asthma were exposed to SHS almost twice as often as children without asthma (23% vs 13% respectively, p<0.0001). Children with asthma exposed to SHS were at higher risk for greater asthma severity (γ = 0.144, p<0.0001)

**Table 2:** Demographics of children with a documented asthma severity enrolled in Easy Breathing and SHS exposure status.

Characteristic	Number (%)	SHS exposed, %	p value^†^
**Asthma**	30163 (25)	23	<0.0001
**Asthma Severity (n = 30,163)**			<0.0001
Intermittent	18774 (57)	20	
Mild Persistent	7528 (23)	22	
Moderate persistent	3633 (11)	26	
Severe persistent	228 (0.7)	3	
**Insurance type**			<0.0001
Medicaid	11888 (39)	30	
Private	14408 (49)	13	
**Area of residence**			<0.0001
Urban core	8394 (28)	28	
Urban periphery	7844 (26)	20	
Rural	2208 (7)	20	
Suburban/wealthy	6791 (23)	10	
**Race/Ethnicity**			<0.0001
Black*	5042 (17)	28	
Hispanic/non Puerto Rican**	2519 (8)	21	
Puerto Rican	7161 (24)	29	
Asian/Pacific Islander	701 (2)	10	
Caucasian	12664 (42)	17	
**Age Group**			<0.0001
0–4 years	10264 (34)	19	
5–9 years	9472 (31)	20	
10–14 years	7679 (26)	22	
15 and older	2748 (9)	31	
**Male**	16653 (55)	20	<0.0001
**Female**	13009 (43)	23	
**Family History**			<0.0001
Yes	20959 (70)	24	
No	7360 (24)	17	
**Eczema**			<0.0001
Yes	5166 (17)	20	
No	13810 (46)	22	
**Cockroach**			<0.0001
Yes	2023 (7)	45	
No	25463 (84)	19	
**Gas stove**			<0.0001
Yes	7399 (25)	30	
No	16043 (53)	19	
**Dog**			0.32
Yes	9489 (32)	23	
No	20674 (67)	21	
**Cat**			<0.0001
Yes	6265 (21)	28	
No	23898 (79)	20	
**Rodent**			0.001
Yes	951 (3)	27	
No	29212 (97)	21	

The % of children with asthma in Easy Breathing uses the entire 120,854 cohort as a denominator (first row), while subsequent rows use the number of children with asthma with a documented severity (n = 30,163) as a denominator. The 30,163 is the analysis cohort for this study.

* Black includes African American and Caribbean/Virgin Islander.

** Hispanic includes those identifying as non-Puerto-Rican Hispanic.

† Indicates complete-case Chi-square analysis for association of individual characteristics with SHS exposure of children with covariates.

Exposure to SHS differed significantly by asthma severity, insurance status, race/ethnicity, and area of residence (Table 2). Black, Hispanic/non-Puerto Rican, and Hispanic/Puerto Rican children with asthma were exposed to SHS more frequently than Caucasian children (OR 2.0, 95% CI 1.8–2.1, OR 1.4, 95% CI 1.3–1.6, OR 2.1, 95% CI 1.9–2.2, respectively and Table 3). Children with asthma living in lower SES communities (i.e., the urban core, urban periphery, and rural areas) were more likely to be exposed to SHS than children living in suburban/wealthy areas (OR 3.6, 95% CI 3.3–4.0, OR 2.2, 95% CI 2.0–2.5, and OR 2.3, 95% CI 2.0–2.6 respectively and Table 3). Children with asthma who were insured by Medicaid were also more likely to be exposed to SHS than privately insured children (OR 3.0, 95% CI 2.8–3.2).

**Table 3:** Risk of exposure to SHS among children with asthma using Logistic Regression with Complete Case analysis.

Ethnicity	OR	95% CI	P value
Caucasian	REF		
Hispanic, non-Puerto Rican	1.414	1.270–1.574	<0.0001
Black	1.988	1.840–2.149	<0.0001
Asian/Pacific Islander	0.561	0.436–0.721	<0.0001
Puerto Rican	2.080	1.939–2.230	<0.0001
**Area of residence**			
Suburban/wealthy	REF		
Urban core	3.625	3.302–3.979	<0.0001
Urban periphery	2.239	2.030–2.469	<0.0001
Rural	2.296	2.012–2.619	<0.0001
**Insurance Status**			
Private	REF		
Medicaid	3.018	2.841–3.207	<0.0001

Values are unadjusted odds ratios (95% CI) relative to no SHS exposure.

SHS exposure, race/ethnicity and area of residence affected asthma severity (Table 4). In unadjusted models, exposure to SHS was associated with an increased risk for persistent asthma as compared to intermittent asthma (S1 Table). Black race, Hispanic/non-Puerto Rican and Puerto Rican ethnicity were also significant predictors of persistent asthma. Children who resided in the urban core experienced the highest risk for persistent asthma. Other risk factors for greater asthma severity included positive family history of asthma, Medicaid insurance, eczema, exposure to cockroaches, and gas stoves (S1 Table). In adjusted models, exposure to SHS remained a significant risk factor for mild and moderate persistent asthma (Table 4). Race/ethnicity remained an overall significant predictor of persistent asthma, with Black, Puerto Rican, and Hispanic/ non-Puerto Rican children at higher risk for moderate persistent asthma than Caucasians. Area of residence remained an overall significant predictor of persistent asthma, with children living in the urban core, urban periphery, and rural areas at higher risk for moderate persistent asthma than children living in suburban/wealthy areas. Children with Medicaid experienced a greater risk for mild and moderate persistent asthma than children with private insurance. Pairwise absolute correlations of risk factors greater than 0.5 are reported (see S7 Table). The highest correlation was reported for Caucasian (1 = Caucasian, 0 = otherwise) and Area of Residence at 0.75. However, the information matrix showed no evidence of ill-conditioning as the largest condition index was only 3.33.

**Table 4:** Main Effects Analysis on Asthma Severity using Multinomial Logistic Regression with Multiple Imputation (N = 30163).

Risk Factor		Mild Persistent (N = 7528)	Moderate Persistent (N = 3633)	Severe Persistent (N = 228)
Family History^b^		**1.22** (1.14,1.31)^b^	**1.49** (1.35,1.64)^b^	1.36 (0.94,1.98)
Medicaid^b^		**1.23** (1.15,1.32)^b^	**1.68** (1.52,1.86)^b^	1.29 (0.89,1.89)
Eczema^b^		**1.14** (1.06,1.22)^b^	**1.25** (1.14,1.37)^b^	**1.47** (1.06,2.04)^a^
Cockroach^b^		**1.18** (1.05,1.32)^b^	**1.46** (1.28,1.66)^b^	**1.50** (1.02,2.20)^a^
SHS^a^		**1.07** (1.00,1.15)^a^	**1.11** (1.02,1.22)^a^	1.22 (0.89,1.65)
Area of residence^b^*	Urban Core^b^	**1.18** (1.06,1.30)^b^	**1.56** (1.34,1.82)^b^	1.60 (0.84,3.03)
	Urban Periphery^b^	**1.22** (1.13,1.33)^b^	**1.30** (1.14,1.49)^b^	1.33 (0.74,2.36)
	Rural^b^	1.06 (0.94,1.19)	**1.46** (1.23,1.74)^b^	1.98 (0.95,4.14)
Race/ Ethnicity^b^**	Hispanic, non-Puerto Rican ^b^	1.06 (0.95,1.19)	**1.35** (1.15,1.57)^b^	1.46 (0.78,2.71)
	Black^b^	**1.12** (1.02,1.23)^a^	**1.26** (1.10,1.43)^b^	1.19 (0.68,2.07)
	Puerto Rican^b^	**1.21** (1.10,1.33)^b^	**1.59** (1.39,1.81)^b^	**2.09** (1.24,3.52)^b^
	Asian/Pacific Islander	1.09 (0.91, 1.31)	0.96 (0.70, 1.32)	1.36 (0.42, 4.44)

Values are adjusted relative risk ratios (95% CI) from multinomial logistic regression models, relative to Intermittent Asthma (N = 18774). The model was adjusted for enrollment date, sex, age, race/ethnicity, family history of asthma, area of residence (SES), type of insurance (Medicaid or private), eczema status, and exposure to dogs, cats, rodents, cockroaches and gas stoves.

*vs Suburban/Wealthy

**vs Caucasian

^a^ p < .05

^b^p < .01.

Superscripts on variable names indicate significance across asthma severity levels (Intermittent vs. Persistent Asthma).

In the multinomial regression model, an interaction between SHS exposure and insurance status was present (p = 0.0325; see S6 Table). S8 Table reports the effects from this interaction analysis. Adjusted odds ratios (aOR) of persistent asthma for insurance-stratified models are presented in Table 5. The crude odds ratios are presented in S2 Table. In adjusted analyses, SHS exposure no longer remained a significant risk factor for persistent asthma among children with Medicaid, whereas children with private insurance exposed to SHS retained an increased risk of persistent asthma (aOR 1.23, 95% CI 1.11–1.37). Puerto Rican children, irrespective of insurance status, experienced high risk for persistent asthma (aOR: 1.20, [1.06,1.36] and aOR: 1.47, [1.28,1.68] for Medicaid and private insurance, respectively).

**Table 5:** Adjusted Odds Ratios (aOR) for Persistent Asthma, Stratified by Insurance Status.

	Medicaid	Private Insurance
	aOR (95%CI)	aOR (95% CI)
No SHS	**REF**	
SHS	1.00 (0.92,1.08)	**1.23** (1.11,1.37)^b^
Race/ethnicity		
Caucasian	**REF**	
Hispanic/non-Puerto Rican	1.05 (0.91,1.21)	**1.18** (1.00,1.38)^a^
Black	1.06 (0.93,1.21)	**1.18** (1.05,1.33)^b^
Asian/Pacific Islander	1.01 (0.72,1.43)	1.06 (0.87,1.30)
Puerto Rican	**1.20** (1.06,1.36)^b^	**1.47** (1.28,1.68)^b^
Area of residence		
Suburban/wealthy	**REF**	
Urban core	**1.35** (1.14,1.60)^b^	**1.25** (1.10,1.42)^b^
Urban periphery	**1.34** (1.13,1.58)^b^	**1.17** (1.07,1.28)^b^
Rural	1.12 (0.90,1.39)	**1.18** (1.04,1.34)^b^

Values are adjusted odds ratios (95% CI) from logistic regression models, relative to Intermittent Asthma (N = 18774). The model was adjusted for enrollment date, sex, age, race/ethnicity, family history of asthma, area of residence (SES), eczema status, and exposure to dogs, cats, rodents, cockroaches and gas stoves.

^a^ p < .05

^b^p < .01.

## Discussion

In this study of children with a physician-confirmed diagnosis and severity of asthma, exposure to SHS was associated with a greater risk for mild and moderate persistent asthma, irrespective of race/ethnicity and area of residence. The risk for persistent asthma with SHS exposure was modified by insurance status but not by race/ethnicity or area of residence, and was only associated with persistent asthma in those with private insurance. The increased risk of persistent asthma with exposure to SHS is particularly important given that U.S. children with asthma have higher rates of SHS exposure compared to children without asthma[2].

Our results suggest a complex relationship between SHS exposure, race/ethnicity, area of residence, poverty (as indicated by Medicaid) and asthma severity. On the one hand, SHS exposure was associated with an overall greater risk for mild and moderate persistent asthma. Children with severe persistent asthma tended to have higher relative risks but the larger standard errors due to small sample sizes rendered the effects statistically insignificant. Hispanic/Puerto Rican, Hispanic/non-Puerto Rican and Black children are more likely to be exposed to SHS than Caucasian children and are more likely to reside in urban areas.

Recent reports highlight the complexities between SHS exposure, race/ethnicity and adverse asthma outcomes. Akinbami et al. reported that SHS exposure was associated with adverse outcomes among non-Hispanic white but not among Black children with asthma[6]. However, Black children without exposure to SHS had similar or greater proportions of adverse outcomes as compared to SHS-exposed Black children and SHS-exposed or unexposed children of other racial or ethnic groups. Because adverse outcomes are associated with asthma severity, our results further support Akinbami’s conclusions. Interestingly, we observed SHS exposure was associated with greater asthma severity among privately insured children even though Medicaid- insured children were more likely to report SHS exposure. It is possible that Medicaid insurance encompasses other uncontrolled factors affecting asthma severity. Hispanic/Puerto Rican, Black, and Hispanic/non-Puerto Rican children are more likely to have Medicaid and more likely to reside in the urban areas where exposures associated with lower housing quality (mold, stress) are more prevalent. Our findings may suggest that for children with asthma living in poverty, the benefits of smoking cessation efforts may be more difficult to demonstrate. A more holistic approach that addresses the economic, environmental and psychosocial factors may be needed for children with persistent asthma living in poverty. Alternatively, it is possible that reporting bias exists as we might expect families of children with asthma to under report exposure as most people know that smoke is bad for children with respiratory disease.

The use of the Easy Breathing data presents several advantages, including guideline-determined asthma severity and the ability to assign unique area of residence categories. Nevertheless, several limitations exist. The prevalence of asthma in Connecticut is not 27% but rather this is the rate of asthma in children screened and enrolled in the Easy Breathing program. Therefore, this sample represents a convenience sample of children already seeking care and may not be representative of all children with asthma. Area of residence was based on town-level characteristics and may not represent the individual household income. Nevertheless, it is useful to group children with a similar area of residence together to make comparisons between children living in different communities, as the Five Connecticuts reflect separate and distinct groups.

Another potential weakness is how exposure to SHS was measured. Misclassification bias may have occurred by parents of children with asthma who are aware of smoking’s adverse effects on asthma. This would have led to a diminished association between SHS and persistent asthma. It is conceivable that parents who enroll in Easy Breathing are more likely to live in smoke-free homes, or underestimate their child’s SHS exposure because of residence in multi-unit housing but this seems unlikely. Another potential weakness was the absence of information on the dose and duration of SHS exposure. Assessing smoke exposures by questionnaires is valid in large-scale epidemiologic studies where biochemical verification is not possible.[18] Overall, self-report of SHS underestimates true smoking prevalence but the sensitivity and cut points of SHS biomarkers (cotinine, nicotine) are highly variable and are influenced by the medium of measurement (salivary vs urine vs serum) and the type of assay used (enzyme-linked immunosorbant assay vs mass spectrometry).SHS biomarkers have not enhanced the quality of smoking data when examining asthma or asthma-related outcomes in a pragmatic setting[17]. Asthma severity in Easy Breathing is based on symptom frequency (impairment domain)[15], and not risk, and may be another limitation. Clinically-determined asthma severity is lower in 36% of children as compared to spirometry-determined severity[31]. This would have resulted in an underestimation of the association between mild and moderate persistent asthma and SHS exposure. Future studies may benefit from including spirometry as an objective factor in determination of asthma severity. It is possible that there was potential bias in asthma diagnosis among the Medicaid—insured children because of reduced access to healthcare, which might have reduced the effect estimates in this population. Lastly, the reliance on self-report of race/ethnicity used in this study and lack of genetic ancestry information can potentially result in misclassification bias. However, Puerto Rican children in this study experienced the highest risks for persistent asthma similar to other studies on the influence of genetic ancestry and asthma risk and lung function [24–26].

SHS exposure is associated with an increased risk for asthma exacerbations and more frequent asthma symptoms. This is the first large-scale study to report an association between exposure to SHS and greater asthma severity using a systematic approach to the classification of asthma severity. Overall, our study demonstrates that exposure to SHS is a significant risk factor for persistent asthma in children. Moreover, we demonstrate that poverty-related sociodemographic factors such as insurance status, but not area of residence or race/ethnicity, modify the association between exposure to SHS and persistent asthma. Future studies should look at the influence of other poverty-related factors. Unfortunately, though SHS exposure among US children without asthma decreased from 1999–2010 there has been no change in exposure to SHS among children with asthma during this period[2]. This work highlights the need for an ecological view of risk for persistent asthma in order to direct research and public health measures effectively and appropriately to manage childhood asthma. Culturally competent tobacco cessation programs delivered by primary care clinicians to parents of children with asthma who smoke may be a key component in reducing disease burden.

## Supporting information

S1 Methods Sensitivity analyses for missing data. (DOCX)

S1 Table Crude Analysis of Asthma Severity using Multinomial Logistic Regression with Multiple Imputation. (DOCX)

S2 Table Crude Odds Ratios (OR) of Persistent Asthma, Stratified by Insurance Status. (DOCX)

S3 Table Missing Information, expressed as Count (%) out of N = 30163 children with documented asthma severity. (DOCX)

S4 Table Main Effects Analysis of Asthma Severity using Multinomial Logistic Regression with Complete Cases (N = 10137). (DOCX)

S5 Table Adjusted Odds Ratios (aOR) for Persistent Asthma, Stratified by Insurance Status using Complete Cases. (DOCX)

S6 Table P-values for Interaction effects of SHS with various risk factors on Asthma Severity from Multinomial Logistic Regression with Multiple Imputation (N = 30163). (DOCX)

S7 Table Pairwise absolute correlations greater than 0.5, computed using complete-case analysis. (DOCX)

S8 Table Effects when including SHS and Medicaid interaction on Asthma Severity using Multinomial Logistic Regression with Multiple Imputation (N = 30163). (DOCX)
