# Supplementary material for: Exposure to secondhand smoke and asthma severity among children in Connecticut
Source: PLoS One. 2017 Mar 31;12(3):e0174541. doi: 10.1371/journal.pone.0174541 (PMC5375151; doi:10.1371/journal.pone.0174541)
Supplement: S1 Methods — (DOCX) [file pone.0174541.s001.docx]

**Supplemental Material: Missing Data**

Supplemental Table 3 displays the amount of missing information for all variables considered in the study. Incomplete data was handled using multiple imputation (MI)^(19)^ , assuming the missing observations were missing at random, with PROC MI/PROC MIANALYZE in SAS version 9.4 (SAS Institute, Cary NC). All default options were used, with the exception of *nimpute* and *minmaxiter*, which were set to 100 and 400, respectively, and fully conditional specification methods were used for categorical variables with more than two categories.

The corresponding complete-case analysis (CCA) results are presented in this Supplement. Specifically, Supplemental Tables 4-5 contain the analogous CCA results to Tables 3-4 based on MI in the main paper. Several sensitivity analyses were also conducted to verify that the estimated MI effects were reasonable, including (1) CCA after setting all missing values to “low risk” values for persistent asthma, (2) CCA after setting all missing values to “high risk” values for persistent asthma, and (3) MI imputing missing values for only potentially confounding variables (Eczema, Gas stove, Cockroach, Family History, Gender, Age) but keeping Area of Residence, Public Insurance, Ethnicity, and SHS missing values as missing (N= 25133).

The overall conclusions from Supplemental Table 4 (CCA) based on N=10137 complete cases were largely consistent with those in Table 3 (MI) based on N=30163 from the main paper, despite the considerably smaller sample size. Notably under CCA, however, SHS does not emerge as a significant risk factor for persistent (mild, moderate or severe) asthma overall in the adjusted main effects analysis (p=0.167), and is only a marginally significant risk factor for mild persistent (vs intermittent) asthma (p=0.053). However, the interaction between SHS and insurance status remained significant under CCA (p=0.0418), as well as in all three sensitivity analyses (p=0.0067. p=0.0251, p=0.0110, respectively). This interaction was investigated further in CCA and sensitivity analyses, and is discussed in the next paragraph. Another notable difference between the CCA and MI results was that Hispanic Non-Puerto Rican ethnicity (as compared to Caucasian ethnicity) was not a significant risk factor for persistent (mild, moderate or severe) asthma overall in CCA. However, in all three sensitivity analyses these effects were similar to those in the MI analysis and significant at the 0.05 level.

The results in Supplemental Table 5 (CCA) largely agree with those in Table 4 (MI) in the main paper for the stratified analysis by insurance status. In particular, SHS remained a significant risk factor for persistent asthma among the privately insured, but not the publicly insured, across all five adjusted analyses (MI, CCA and three sensitivity analyses). Two notable differences in the CCA results were that (i) Puerto Rican ethnicity (as compared to Caucasian ethnicity) was not a significant risk factor for persistent asthma among publicly insured in CCA and (ii) Hispanic (Non-Puerto-Rican) ethnicity and Black race (as compared to Caucasian ethnicity) were not significant risk factors for persistent asthma among privately insured. For (i), however, all three sensitivity analyses yielded the same conclusion as the MI analysis that Puerto Rican ethnicity (as compared to Caucasian ethnicity) is a significant risk factor for persistent asthma among publicly insured (p=0.0137, p= 0.0026, p<0.0001, respectively). For (ii), again all three sensitivity analyses yielded the same conclusion as the MI analysis that Hispanic Non-Puerto Rican ethnicity (as compared to Caucasian ethnicity) is a significant risk factor for persistent asthma among privately insured (p=0.0323, p= 0.0044, p=0.0003, respectively), while only sensitivity analyses (2) and (3) found Black race to be a significant risk factor among privately insured at the 0.05 level (p=0.0026, p=0.0029, respectively). The “low risk” sensitivity analysis (1) provided a reasonably low p-value for this effect (p=0.0631). Based on these results, we feel the conclusions from MI presented in the main paper are justifiable.
