# Supplementary material for: Exposure to secondhand smoke and asthma severity among children in Connecticut
Source: PLoS One. 2017 Mar 31;12(3):e0174541. doi: 10.1371/journal.pone.0174541 (PMC5375151; doi:10.1371/journal.pone.0174541)
Supplement: S1 Table — (DOCX) [file pone.0174541.s002.docx]

| **Supplemental Table 1. Crude Analysis of Asthma Severity using Multinomial Logistic Regression with Multiple Imputation (N=30163)** | | | | |
| --- | --- | --- | --- | --- |
| **Risk Factor** |  | **Mild Persistent (N=7528)** | **Moderate Persistent (N=3633)** | **Severe Persistent (N=228)** |
| Year of enrollment^b^ |  | **0.97** (0.96,0.98)^b^ | **0.97** (0.96,0.97)^b^ | **0.82** (0.79,0.85)^b^ |
| Age^b^ |  | **0.96** (0.96,0.97)^b^ | **1.01** (1.00,1.02)^a^ | 1.02 (0.99,1.05) |
| Family History^b^ |  | **1.37** (1.28,1.46)^b^ | **1.92** (1.74,2.11)^b^ | **1.96** (1.37,2.82)^b^ |
| Gender^a^ |  | **1.08** (1.02,1.14)^b^ | 1.00 (0.93,1.07) | 1.07 (0.82,1.40) |
| Public Insurance^b^ |  | **1.51** (1.43,1.60)^b^ | **2.63** (2.43,2.86)^b^ | **2.82** (2.10,3.80)^b^ |
| Gas Stove^b^ |  | **1.11** (1.04,1.18)^b^ | **1.47** (1.36,1.60)^b^ | **1.65** (1.23,2.21)^b^ |
| Eczema^b^ |  | **1.14** (1.06,1.22)^b^ | **1.17** (1.07,1.28)^b^ | 1.29 (0.93,1.78) |
| Cockroach^b^ |  | **1.53** (1.38,1.70)^b^ | **2.48** (2.20,2.78)^b^ | **3.68** (2.60,5.20)^b^ |
| Rodent |  | 0.92 (0.79,1.07) | **0.75** (0.60,0.93)^a^ | 0.65 (0.27,1.59) |
| Dog^b^ |  | **0.82** (0.77,0.87)^b^ | **0.77** (0.71,0.84)^b^ | **0.72** (0.53,0.97)^a^ |
| Cat^b^ |  | **0.90** (0.84,0.96)^b^ | **0.71** (0.65,0.79)^b^ | **0.56** (0.38,0.82)^b^ |
| SHS^b^ |  | **1.21** (1.13,1.29)^b^ | **1.53** (1.41,1.66)^b^ | **1.98** (1.49,2.63)^b^ |
| Area of residence^b^* | Urban Core^b^ | **1.69** (1.57,1.82)^b^ | **3.35** (2.99,3.74)^b^ | **5.04** (3.15,8.06)^b^ |
|  | Urban Periphery^b^ | **1.37** (1.27,1.48)^b^ | **1.73** (1.53,1.96)^b^ | 1.69 (0.98,2.91) |
|  | Rural^b^ | 1.09 (0.97,1.22) | **1.63** (1.37,1.93)^b^ | 1.77 (0.85,3.67) |
| Race/ Ethnicity^b^** | Hispanic/non-Puerto Rican^b^ | **1.33** (1.20,1.47)^b^ | **2.18** (1.91,2.49)^b^ | **2.46** (1.46,4.15)^b^ |
|  | Black^b^ | **1.42** (1.31,1.53)^b^ | **2.09** (1.88,2.32)^b^ | **2.37** (1.54,3.66)^b^ |
|  | Puerto Rican^b^ | **1.65** (1.54,1.77)^b^ | **3.12** (2.84,3.42)^b^ | **5.32** (3.78,7.50)^b^ |
|  | Asian/Pacific Islander | 1.10 (0.92,1.32) | 0.96 (0.71,1.31) | 1.14 (0.35,3.69) |

Values are crude relative risk ratios (95% CI) from multinomial logistic regression models, relative to Intermittent Asthma (N=18774). *vs Suburban/Wealthy, **vs Caucasian, ^a^ p<.05, ^b^p<.01. Superscripts on variable names indicate significance across asthma severity levels (Intermittent vs. Persistent Asthma).
