# Supplementary material for: Exposure to secondhand smoke and asthma severity among children in Connecticut
Source: PLoS One. 2017 Mar 31;12(3):e0174541. doi: 10.1371/journal.pone.0174541 (PMC5375151; doi:10.1371/journal.pone.0174541)
Supplement: S2 Table — (DOCX) [file pone.0174541.s003.docx]

| Supplemental Table 2.  Crude Odds Ratios (OR) of Persistent Asthma, Stratified by Insurance Status | | |
| --- | --- | --- |
|  | Public Insurance | Private Insurance |
|  | OR (95%CI) | OR (95% CI) |
| No SHS | **REF** | |
| SHS | 1.06 (0.99,1.15) | **1.31** (1.19,1.45)^b^ |
| Race/ethnicity | | |
| Caucasian | **REF** | |
| Hispanic/non-Puerto Rican | **1.23** (1.08,1.39)^b^ | **1.32** (1.14,1.54)^b^ |
| Black | **1.26** (1.13,1.41)^b^ | **1.41** (1.27,1.56)^b^ |
| Asian/Pacific Islander | 0.97 (0.69,1.35) | 1.08 (0.89,1.32) |
| Puerto Rican | **1.51** (1.37,1.66)^b^ | **1.85** (1.65,2.07)^b^ |
| Area of residence | | |
| Suburban/wealthy | **REF** | |
| Urban Core | **1.64** (1.42,1.91)^b^ | **1.72** (1.56,1.90)^b^ |
| Urban Periphery | **1.39** (1.18,1.63)^b^ | **1.28** (1.18,1.39)^b^ |
| Rural | 1.09 (0.88,1.35) | **1.17** (1.03,1.31)^a^ |

Values are adjusted odds ratios (95% CI) from logistic regression models, relative to Intermittent Asthma. ^a^ p<.05, ^b^p<.01.
