# Supplementary material for: Exposure to secondhand smoke and asthma severity among children in Connecticut
Source: PLoS One. 2017 Mar 31;12(3):e0174541. doi: 10.1371/journal.pone.0174541 (PMC5375151; doi:10.1371/journal.pone.0174541)
Supplement: S3 Table — (DOCX) [file pone.0174541.s004.docx]

| Supplemental Table 3: Missing Information, expressed as Count (%) out of N=30163 children with documented asthma severity. | |
| --- | --- |
| Eczema | 11187 (37.0) |
| Gas stove | 6721 (22.3) |
| Area of Residence | 4926 (16.3) |
| Public Insurance | 3867 (12.8) |
| Cockroach | 2677 (8.8) |
| Ethnicity | 2076 (6.9) |
| SHS | 2018 (6.7) |
| Family History | 1844 (6.1) |
| Gender | 501 (1.7) |
| Age | 54 (0.2) |
| Enrollment Year | 0 (0.0) |
| Dog | 0 (0.0) |
| Cat | 0 (0.0) |
| Rodent | 0 (0.0) |
