# Supplementary material for: Exposure to secondhand smoke and asthma severity among children in Connecticut
Source: PLoS One. 2017 Mar 31;12(3):e0174541. doi: 10.1371/journal.pone.0174541 (PMC5375151; doi:10.1371/journal.pone.0174541)
Supplement: S4 Table — (DOCX) [file pone.0174541.s005.docx]

| **Supplemental Table 4. Main Effects Analysis of Asthma Severity using Multinomial Logistic Regression with Complete Cases (N=10137)** | | | | |
| --- | --- | --- | --- | --- |
| **Risk Factor** |  | **Mild Persistent (N=2495)** | **Moderate Persistent (N=1176)** | **Severe Persistent (N=33)** |
| Enroll Year^b^ |  | **0.97** (0.95,0.98)^b^ | **0.97** (0.95,0.99)^b^ | **0.85** (0.76,0.96)^b^ |
| Age^b^ |  | **0.96** (0.95,0.97)^b^ | **1.02** (1.01,1.03)^b^ | 1.02 (0.94,1.10) |
| Family History^b^ |  | **1.29** (1.15,1.44)^b^ | **1.79** (1.50,2.13)^b^ | 1.34 (0.54,3.33) |
| Gender |  | 1.04 (0.94,1.14) | 1.06 (0.93,1.21) | 0.992 (0.50,1.98) |
| Public Insurance^b^ |  | **1.28** (1.15,1.44)^b^ | **1.88** (1.62,2.19)^b^ | 0.61 (0.27,1.39) |
| Gas Stove |  | 0.96 (0.87,1.08) | 1.05 (0.91,1.21) | 1.13 (0.53,2.42) |
| Eczema^b^ |  | **1.24** (1.12,1.38)^b^ | **1.39** (1.21,1.60)^b^ | **2.06** (1.01,4.21)^a^ |
| Cockroach^b^ |  | **1.37** (1.08,1.74)^b^ | **1.50** (1.15,1.96)^b^ | **3.35** (1.12,9.90)^a^ |
| Rodent |  | 0.99 (0.75,1.29) | 1.02 (0.70,1.47) | <0.001 (<0.001, >999.99) |
| Dog |  | 0.94 (0.85,1.05) | 1.08 (0.93,1.24) | 1.17 (0.55,2.50) |
| Cat |  | 1.06 (0.94,1.19) | 0.97 (0.82,1.14) | 0.61 (0.23,1.68) |
| SHS |  | 1.13 (1.00,1.28) | 1.06 (0.90,1.24) | 0.59 (0.22,1.59) |
| Area of residence^b^* | Urban Core^b^ | 1.10 (0.94,1.30) | **1.54** (1.22,1.94)^b^ | **7.22** (1.32,39.43)^a^ |
|  | Urban Periphery^b^ | **1.19** (1.04,1.36)^b^ | **1.28** (1.04,1.57)^a^ | 4.19 (0.85,20.58) |
|  | Rural^b^ | 1.13 (0.94,1.35) | **1.71** (1.32,2.21)^b^ | **12.39** (2.46,62.46)^b^ |
| Race/ Ethnicity^a^** | Hispanic, non-Puerto Rican | 0.97 (0.80,1.17) | 1.20 (0.93,1.55) | 1.06 (0.26,4.33) |
|  | Black^b^ | 1.05 (0.90,1.23) | **1.42** (1.15,1.76)^b^ | 0.43 (0.10,1.82) |
|  | Puerto Rican^b^ | 1.12 (0.96,1.31) | **1.50** (1.21,1.85)^b^ | 1.68 (0.55,5.16) |
|  | Asian/Pacific Islander | 0.99 (0.74, 1.33) | 0.94 (0.58, 1.54) | <0.001 (<0.001, >999.99) |

Values are adjusted relative risk ratios (95% CI) from multinomial logistic regression models, relative to Intermittent Asthma (N=6433). The model was adjusted for enrollment date, sex, age, race/ethnicity, family history of asthma, area of residence (SES), type of insurance (public or private), eczema status, and exposure to dogs, cats, rodents, cockroaches and gas stoves. *vs Suburban/Wealthy, **vs Caucasian, ^a^ p<.05, ^b^p<.01. Superscripts on variable names indicate significance across asthma severity levels (Intermittent vs. Persistent Asthma).
