# Supplementary material for: Exposure to secondhand smoke and asthma severity among children in Connecticut
Source: PLoS One. 2017 Mar 31;12(3):e0174541. doi: 10.1371/journal.pone.0174541 (PMC5375151; doi:10.1371/journal.pone.0174541)
Supplement: S5 Table — (DOCX) [file pone.0174541.s006.docx]

| Supplemental Table 5.  Adjusted Odds Ratios (aOR) for Persistent Asthma, Stratified by Insurance Status using Complete Cases | | |
| --- | --- | --- |
|  | Public Insurance (N=5052) | Private Insurance (N=6507) |
|  | aOR (95%CI) | aOR (95% CI) |
| No SHS | **REF** | |
| SHS | 0.98 (0.86,1.11) | **1.29** (1.11,1.51)^b^ |
| Race/ethnicity | | |
| Caucasian | **REF** | |
| Hispanic/non-Puerto Rican | 1.03 (0.83,1.28) | 1.11 (0.88,1.39) |
| Black | 1.06 (0.88,1.28) | 1.16 (0.97,1.39) |
| Asian/Pacific Islander | 1.04 (0.63,1.71) | 0.97 (0.72,1.30) |
| Puerto Rican | 1.08 (0.90,1.30) | **1.49** (1.22,1.80)^b^ |
| Area of residence | | |
| Suburban/wealthy | **REF** | |
| Urban core | **1.30** (1.03,1.64)^a^ | **1.20** (1.00,1.45)^a^ |
| Urban periphery | **1.36** (1.08,1.70)^b^ | **1.15** (1.00,1.31)^a^ |
| Rural | 1.21 (0.92,1.61) | **1.41** (1.17,1.70)^b^ |

Values are adjusted odds ratios (95% CI) from logistic regression models, relative to Intermittent Asthma. The model was adjusted for enrollment date, sex, age, race/ethnicity, family history of asthma, area of residence (SES), eczema status, and exposure to dogs, cats, rodents, cockroaches and gas stoves., ^a^ p<.05, ^b^p<.01.
