# Supplementary material for: Exposure to secondhand smoke and asthma severity among children in Connecticut
Source: PLoS One. 2017 Mar 31;12(3):e0174541. doi: 10.1371/journal.pone.0174541 (PMC5375151; doi:10.1371/journal.pone.0174541)
Supplement: S6 Table — (DOCX) [file pone.0174541.s007.docx]

Supplemental Table 6. P-values for Interaction effects of SHS with various risk factors on Asthma Severity from Multinomial Logistic Regression with Multiple Imputation (N=30163).

| **Interaction Term** | **Degrees of Freedom** | **P-value** |
| --- | --- | --- |
| Age*SHS | 3 | 0.7387 |
| Area of Residence*SHS | 9 | 0.3836 |
| Enroll Year*SHS | 3 | 0.4854 |
| Ethnicity*SHS | 12 | 0.3281 |
| Gender*SHS | 3 | 0.7576 |
| Medicaid*SHS | 3 | 0.0325 |

Degrees of freedom and p-values are from interaction tests under multinomial logistic regression models, relative to Intermittent Asthma (N=18774). A separate model, adjusted for enrollment date, sex, age, race/ethnicity, family history of asthma, area of residence (SES), type of insurance (Medicaid or private), eczema status, and exposure to dogs, cats, rodents, cockroaches and gas stoves, was fit for each interaction term considered.
