# Supplementary material for: Exposure to secondhand smoke and asthma severity among children in Connecticut
Source: PLoS One. 2017 Mar 31;12(3):e0174541. doi: 10.1371/journal.pone.0174541 (PMC5375151; doi:10.1371/journal.pone.0174541)
Supplement: S7 Table — (DOCX) [file pone.0174541.s008.docx]

Supplemental Table 7. Pairwise absolute correlations greater than 0.5, computed using complete-case analysis.

| **Risk Factors** | **Absolute Correlation*** |
| --- | --- |
| Caucasian, Area of Residence | 0.7498 |
| Caucasian, Cockroach | 0.7185 |
| Caucasian, Medicaid | 0.7128 |
| Area of Residence, Cockroach | 0.6483 |
| Medicaid, Cockroach | 0.6382 |
| Caucasian, Cat | 0.5020 |

*Only polychoric correlations were greater than 0.5.
