# Supplementary material for: Exposure to secondhand smoke and asthma severity among children in Connecticut
Source: PLoS One. 2017 Mar 31;12(3):e0174541. doi: 10.1371/journal.pone.0174541 (PMC5375151; doi:10.1371/journal.pone.0174541)
Supplement: S8 Table — (DOCX) [file pone.0174541.s009.docx]

**Supplemental Table 8. Effects when including SHS and Medicaid interaction on Asthma Severity using Multinomial Logistic Regression with Multiple Imputation (N=30163).**

| **Risk Factor** |  | **Mild Persistent (N=7528)** | **Moderate Persistent (N=3633)** | **Severe Persistent (N=228)** |
| --- | --- | --- | --- | --- |
| Enroll Year^b^ |  | **0.98** (0.98,0.99)^b^ | 1.00 (0.99,1.01) | **0.87** (0.84,0.90)^b^ |
| Age^b^ |  | **0.97** (0.96,0.97)^b^ | **1.02** (1.01,1.03)^b^ | 1.03 (1.00,1.06) |
| Family History^b^ |  | **1.22** (1.15,1.31)^b^ | **1.49** (1.35,1.64)^b^ | 1.37 (0.94,1.98) |
| Gender |  | **1.06** (1.00,1.12)^a^ | 1.04 (0.96,1.12) | 1.12 (0.85,1.46) |
| Medicaid^b^ |  | **1.30** (1.20,1.40)^b^ | **1.75** (1.57,1.95)^b^ | 1.36 (0.88,2.09) |
| Gas Stove |  | 0.97 (0.91,1.04) | 1.08 (0.99,1.18) | 1.06 (0.78,1.44) |
| Eczema^b^ |  | **1.14** (1.06,1.22)^b^ | **1.25** (1.14,1.37)^b^ | **1.47** (1.06,2.04)^a^ |
| Cockroach^b^ |  | **1.19** (1.06,1.33)^b^ | **1.47** (1.29,1.67)^b^ | **1.50** (1.02,2.21)^a^ |
| Rodent |  | 1.05 (0.90,1.23) | 0.90 (0.72,1.14) | 0.79 (0.32,1.95) |
| Dog |  | 0.96 (0.91,1.03) | 0.99 (0.91,1.08) | 1.06 (0.77,1.45) |
| Cat |  | 1.05 (0.98,1.12) | 0.93 (0.84,1.03) | 0.81 (0.54,1.21) |
| SHS^b^ |  | **1.22** (1.09,1.37)^b^ | **1.23** (1.03,1.45)^a^ | 1.36 (0.75,2.46) |
| Area of residence^b^* | Urban Core^b^ | **1.17** (1.05,1.29)^b^ | **1.55** (1.33,1.81)^b^ | 1.58 (0.83,3.01) |
|  | Urban Periphery^b^ | **1.21** (1.12,1.32)^b^ | **1.30** (1.13,1.48)^b^ | 1.32 (0.74,2.35) |
|  | Rural^b^ | 1.05 (0.93,1.18) | **1.45** (1.22,1.73)^b^ | 1.96 (0.94,4.11) |
| Race/ Ethnicity^b^** | Hispanic, non-Puerto Rican ^b^ | 1.06 (0.94,1.19) | **1.34** (1.15,1.56)^b^ | 1.45 (0.78,2.69) |
|  | Black^b^ | **1.12** (1.02,1.23)^a^ | **1.25** (1.10,1.43)^b^ | 1.18 (0.68,2.06) |
|  | Puerto Rican^b^ | **1.20** (1.09,1.32)^b^ | **1.58** (1.39,1.80)^b^ | **2.08** (1.23,3.51)^b^ |
|  | Asian/Pacific Islander | 1.09 (0.91, 1.31) | 0.96 (0.70, 1.32) | 1.36 (0.42, 4.45) |
| Medicaid*SHS^a^ |  | **0.81** (0.69, 0.94)^b^ | 0.86 (0.70, 1.05) | 0.84 (0.41, 1.70) |

Values are adjusted relative risk ratios (95% CI) from multinomial logistic regression models, relative to Intermittent Asthma (N=18774). The model was adjusted for enrollment date, sex, age, race/ethnicity, family history of asthma, area of residence (SES), type of insurance (Medicaid or private), eczema status, and exposure to dogs, cats, rodents, cockroaches and gas stoves. *vs Suburban/Wealthy, **vs Caucasian, ^a^ p<.05, ^b^p<.01. Superscripts on variable names indicate significance across asthma severity levels (Intermittent vs. Persistent Asthma).
